# Silver nanoparticles as a medical device in healthcare settings: a five-step approach for candidate screening of coating agents

**DOI:** 10.1098/rsos.171113

**Published:** 2018-01-31

**Authors:** Valentina Marassi, Luisana Di Cristo, Stephen G. J. Smith, Simona Ortelli, Magda Blosi, Anna L. Costa, Pierluigi Reschiglian, Yuri Volkov, Adriele Prina-Mello

**Affiliations:** 1 Department of Chemistry ‘G. Ciamician’, Via Selmi 2, 40126 Bologna, Italy; 2 Department of Clinical Medicine, Trinity Translational Medicine Institute (TTMI), School of Medicine, Trinity College, Dublin 8, Republic of Ireland; 3 Department of Clinical Microbiology, Sir Patrick Dun Research Laboratory, School of Medicine, Trinity College, Dublin 8, Republic of Ireland; 4 Institute of Science and Technology for Ceramics (CNR-ISTEC), National Research Council of Italy, Via Granarolo 64, 48018 Faenza, RA, Italy; 5 AMBER Centre and CRANN Institute, Trinity College Dublin, Dublin 2, Republic of Ireland

**Keywords:** silver nanoparticles, healthcare, coating agents, hollow-fibre flow-field flow fractionation, antimicrobials, cellular toxicity

## Abstract

Silver nanoparticle-based antimicrobials can promote a long lasting bactericidal effect without detrimental toxic side effects. However, there is not a clear and complete protocol to define and relate the properties of the particles (size, shape, surface charge, ionic content) with their specific activity. In this paper, we propose an effective multi-step approach for the identification of a ‘purpose-specific active applicability window’ to maximize the antimicrobial activity of medical devices containing silver nanoparticles (Ag NPs) (such as surface coaters), minimizing any consequent risk for human health (safety by design strategy). The antimicrobial activity and the cellular toxicity of four types of Ag NPs, differing in their coating composition and concentration have been quantified. Through the implementation of flow-field flow fractionation, Ag NPs have been characterized in terms of metal release, size and shape. The particles are fractionated in the process while being left unmodified, allowing for the identification of biological particle-specific contribution. Toxicity and inflammatory response *in vitro* have been assessed on human skin models, while antimicrobial activity has been monitored with both non-pathogenic and pathogenic *Escherichia coli*. The main benefit associated with such approach is the comprehensive assessment of the maximal effectiveness of candidate nanomaterials, while simultaneously indexing their properties against their safety.

## 1. Background

The production of auto-sanitizing products for healthcare is highly desirable given the increasing incidence of healthcare-associated infection (HCAIs) [1]. The use of nanoparticles as active components in composite materials in place of conventional chemical products such as ethanol or bleach can guarantee long lasting bactericidal effects while not being toxic to the human body. Silver nanoparticles (Ag NPs) are known for their antimicrobial applications in common/household items, and their use in commercial products is increasing. In fact, they are already widely found as antiseptic additives in packaging, fabric, and are also ideal candidates as additives for tile coatings [2]. The global Ag NP market is indeed expected to reach $2.45 billion by 2022, with increasing demand for antimicrobial materials in healthcare applications [3]. Healthcare is the largest sector of that market, accounting for over 30% of the global Ag NP market revenue in 2014. With the pressing need to prevent HCAIs, it is expected that the use of Ag NPs in medical devices, equipment and textiles will further expand. The increased usage of nanoparticle-based medical devices has raised the attention of the European and International regulators and occupational safety community [4–6], leading to new guidelines for safety assessment of nanotechnology-enabled medical devices [7]. Ag NPs are thought to exert their antimicrobial effect through the release of free metal Ag^+^ ions. Indeed, silver ions are powerful antimicrobials themselves, but they are easily sequestered by chloride, phosphate, proteins and other cellular components. However, Ag NPs are less susceptible to sequestration and are thus a more effective delivery method [8]. The biological effect of the nanoparticles is largely unproven, but recent results supported the theory that the cytotoxic effects of nanosilver are a combination of precipitated silver complexes and organic silver compounds rather than free silver ions [9]. It is suggested that the antibacterial activity is owing to the generation of silver ions in the aqueous solution binding with the proteins on the bacteria cell membrane and inhibiting cell respiration and reproduction [10–12]. Particles size, free surface area, shape and charge will affect the bioavailability of ions in terms of dissolution or transport and interaction with biological targets [13–15]. Toxicity refers to any deleterious effects on an organism upon exposure to silver. Obviously, if the practical intent is to disinfect or sterilize a specific type of organism, then toxicity may be interpreted as a positive outcome (e.g. antibacterial, antiviral, etc.). However, if the same material exerts unintended or undesired impacts to other organisms, then such toxicity may be interpreted as a potential hazard [1–16]. An ideal antimicrobial candidate, therefore, needs to be selectively toxic, i.e. it is antibacterial at a given concentration but not toxic to humans. For a realistic evaluation of risk/benefit ratio, the comparison between human toxicity and antimicrobial effect has to be considered in terms of exposure times which may differ considerably, [14] e.g. a treated inanimate surface (such as a wall) is unlikely to have a long human exposure time (contact) while the antiseptic effect can be evaluated over a longer period. Concerns have been raised currently regarding the potential toxicities of Ag NPs [17]. For example, the Scientific Committee on Emerging and Newly Identified Health Risks (SCENIHR committees) [18] highlighted the importance of considering the different forms of silver used in consumer and medical products, because Ag NPs undergo several transformations as aggregation, agglomeration, dissolution and subsequent speciation. The chemical species that are actually present determine the bioavailability and toxicity of silver in the environment. Focusing on medical devices, the ‘Guidance on the Determination of Potential Health Effects of Nanomaterials Used in Medical Devices', where nanosilver is widely mentioned, addresses the specific aspects that need to be considered in the safety evaluation of nanomaterials. This guidance highlights the need for special considerations in relation to the safety evaluation of nanomaterials, in view of the possible distinct properties, interactions, and effects that may differ from conventional forms of the same materials [7]. It recommends a stepwise approach where the first step is the chemical identification and characterization of nanomaterials used in the production of a medical device [19]. Relevant methods for nanomaterial characterization may include size separation and extraction and chemical analysis/detection by spectroscopic or mass spectrometric techniques [7]. The European NanoSafety Cluster has also come forward by suggesting a multi-step approach based on the implementation of the three classes of characterization techniques: imaging-based, light scattering-based and separation-based. Such platform should be able to measure nanoparticle primary size, the size distribution in complex matrices, while providing information on different populations present and their surface properties [20]. To address all these issues and identify the purpose-specific applicability window of Ag NPs we propose a five-step approach based first on separation (through flow-field flow fractionation, FlFFF) and characterization of particles, obtaining information about the dimension, the shape and the effective coating of particles, and quantifying the initial free ion presence. FlFFF has been used widely to characterize, concentrate and quantify ion release of engineered nanoparticles, especially when combined with inductively coupled plasma-mass spectrometry (ICP-MS) [21] and has been also employed to evaluate protein corona onto Ag NPs [22,23]. By exploiting hollow fibre flow-field flow fractionation (HF5), the commercial miniaturized version of FlFFF, it has been also possible to collect fractions of purified particles, without destroying their colloidal properties as more traditional ultrafiltration systems do. Secondly, to rapidly and effectively study the antimicrobial effect of the particles over time, luminescent bacteria were used: luminescence is directly proportional to viability thanks to a plasmid modification thus reducing analysis time [24]. The third step involved *in vitro* toxicity tests on skin models. Further to that isolated particles have been tested for their antimicrobial activity and cellular toxicity to quantify particle-specific contribution. Finally, all the parameters were gathered together for each preparation of Ag NPs, in order to select the best antimicrobial candidate and the set of physicochemical properties required of silver nanomaterials to be used within medical devices in healthcare settings.

## 2. Material and methods

### 2.1. Reagents

Foetal bovine serum (FBS) and culture media were purchased from Sigma-Aldrich (Dublin, Ireland). Calcein was purchased from Molecular Probes, Invitrogen (Dublin, Ireland). ThermoFisher (Dublin, Ireland) was the source of all the other chemicals, whenever not specified otherwise.

### 2.2. Silver nanoparticles

For this study, four different Ag NP suspensions have been employed. Ag Pristine (0.02 wt% silver concentration) was provided by Colorobbia SpA (Italy). Polyvinylpyrrolidone-coated sample (Ag PVP, 0.02 wt% silver concentration), citrate-coated sample (Ag CIT, 0.02 wt% silver concentration) and hydroxyethyl cellulose-coated sample (Ag HEC, 0.02 wt% silver concentration) were synthesized in ISTEC-CNR (Faenza, Italy). Pristine and Ag PVP were obtained within the same process on a different scale (industrial scale for the Pristine, laboratory scale for the PVP coated material) using the same reducing (glucose) and capping (polyvinylpyrrolidone) agents but an excess of polyvinylpyrrolidone for the industrial scale synthesis [25]. Ag CIT was obtained using sodium citrate (Sigma-Aldrich) both as reducing agent and as a stabilizer, starting from AgNO_3_ solution. The synthesis reaction occurred in basic environmental and at 70°C by microwave heating, which enables homogeneous heating and rapid achievement of the desired temperatures [26]. The Ag HEC was synthesized, at room temperature, reducing a solution of AgNO_3_ by hydroxyethyl cellulose, which was also used as the capping agent. The reduction synthesis was catalysed by NaOH (Sigma-Aldrich) [27].

### 2.3. Hollow-fibre flow-field flow fractionation (HF5)

HF5 analyses were performed using an Agilent 1200 HPLC system (Agilent Technologies, Santa Clara, CA, USA) complete with degasser, autosampler, isocratic pump and an Agilent 1100 diode array detector (DAD) UV/Vis spectrophotometer combined with an Eclipse^®^ DUALTEC separation system (Wyatt Technology Europe, WTE, Dernbach, Germany). The HF5 channel (Wyatt Technology Europe) consisted of the commercial PES fibre and cartridge provided by WTE. Detailed description of the system was reported in a previous work published by some of the co-authors [28]. The software ChemStation version B.04.02 (Agilent Technologies) and Wyatt Eclipse @ ChemStation version 3.5.02 (Wyatt Technology Europe) were used to handle the separation parameters. An 18-angle multi-angle light scattering (MALS) detector model DAWN HELEOS (Wyatt Technology Corporation, Santa Barbara, CA, USA) operating at a wavelength of 658 nm, was used to measure compute the *R*_g_ of particles in solution and was handled with ASTRA^®^ software v. 5.3.2.14 (Wyatt Technology Corporation). An HF5 method is composed of four steps: focus, focus–injection, elution and elution–injection. During focus the mobile phase is split into two different streams entering from the fibre's inlet and outlet; during focus–injection, the sample is introduced through the inlet and focalized in a narrow band. During the elution step, the inlet flow splits in two: the longitudinal flow (*V*_c_, going to the detectors) and the crossflow (*V*_x_, determining the applied field). Lastly, the crossflow is released and the stream of mobile phase passes through the injection line to clean it before the next injection. The flow conditions for the different HF5 analysis are shown in table 1. A volume of Ag NPs of 5 µl was injected for the characterization of the sample in order to avoid saturation of the scattering signal, while a volume of Ag NPs of 100 µl was injected to collect both the ionic fraction and the isolated nanoparticles. When particles travelling in a parabolic flow profile are subjected to a crossflow, they localize at different points of the flow profile, according to their diffusion coefficient *D* (proportional to their hydrodynamic radius *R*_h_). Smaller particles experience a higher flow rate and faster elution (normal mode). The *R*_h_ can be obtained from direct calculation or through calibration with standards of known size. The FlFFF theory behind this work is described in the previous literature [7–29]. When the analysed particles are not spherical, *R*_h_ is an estimation of the equivalent radius of a sphere with the same coefficient of diffusion *D*. On the other hand, the *R*_g_ value provided by the MALS gives information about the compactness on the particles: two particles with same hydrodynamic radius (*R*_h_), but with different *R*_g_ values, may have a different mass distribution, and thus, different shapes. Combining the two sizing techniques via *R*_g_/*R*_h_ ratios, a shape factor is obtained, reflecting the compactness and shape of the particles. For example, this corresponds to a value of 0.77 for a compact sphere and increases to about 4 for needle-like conformation, or decreases to about 0.6/0.5 for particles presenting a hard core and a soft shell/coating.

**Table 1.** Flow conditions for HF5 analyses. (*V*_c_, longitudinal flow; *V*_x_*,* cross/focus flow.)

steps (versus) method	focus (ml min^−1^)	focus–injection (ml min^−1^)	elution (ml min^−1^)	elution–injection (ml min^−1^)
particle characterization	*V*_c_ = 0.35	*V*_c_ = 0.35	*V*_c_ = 0.35	*V*_c_ = 0.35
	*V*_x_ = 0.80	*V*_x_ = 0.80	*V*_x_ = 0.1	*V*_x_ = 0.0
	time= 2 min	time = 3 min	time = 10 min	time = 3 min
cationic Ag collection and fraction collection	*V*_c_ = 0.35	*V*_c_ = 1.0	*V*_c_ = 0.35	*V*_c_ = 0.35
	*V*_x_ = 0.80	*V*_x_ = 0.8	*V*_x_ = 0.1	*V*_x_ = 0.0
	time = 0.5 min	time = 20 min	time = 10 min	time = 3 min

### 2.4. Ag^+^/Ag determination

The Ag^+^/Ag ratio was determined in Milli-Q water, analysing samples through an ad hoc analytical method listed in table 2, able to retain Ag NPs and filtrate away Ag^+^ ions. The proof of concept of the efficacy of this method has already been demonstrated in a previous work [30] employing flame absorption atomic spectroscopy (FAAS). The subsequent quantification of ionic Ag with FAAS was used to estimate the Ag^+^/Ag ratio. This is a non-destructive method, allowing for the collection of filtered nanoparticles that can be, therefore, tested individually to investigate particle-specific activity. In this work, because the preliminary determination of Ag^+^ through FAAS showed a very low concentration of silver, graphite furnace was used instead. To quantify the ionic fraction a volume of 10 ml was collected, 1 ml of concentrated HNO_3_ was added and the concentration of Ag^+^ was determined with atomic absorption spectroscopy by interpolation on a standard calibration curve (LoD = 0.2 ppb) following opportune dilution. Pd and Mn(NO_3_)_2_ were used as modifiers to prevent analyte loss. Each quantitative analysis was repeated three times and the ionic silver amount, expressed as percentage on the total silver content, is listed in table 2. By subtraction, the amount of ‘nano’ silver present in the collected nanoparticles was obtained and by correlating it with the fraction volume the subsequent concentration was determined. The scheme of such a procedure divided in steps is further detailed in the electronic supplementary information.

**Table 2.** Physico-chemical properties of Ag NPs. (*R*_h_, hydrodynamic radius determined by NTA; *R*_g_, radius of gyration determined by MALS; *ζ*, zeta potential.)

measured parameters (versus) Ag NPs	*R*_h_ (nm)	*R*_g_ (nm)	shape factor *R_g_*/*R_h_*	*ζ*_H2O_ (mV)	*ζ*_DMEM_ (mV)	% Ag^+^ (w/w)
Pristine	33.0 ± 2.5	18.5 ± 0.15	0.55	−13.0 ± 9.0	−7.61 ± 0.05	3.87 ± 0.08
Ag PVP	19.0 ± 3.6	13.5 ± 0.39	0.73	−25.0 ± 9.0	−6.99 ± 0.05	3.67 ± 0.09
Ag CIT	28.0 ± 2.9	13.6 ± 0.25	0.49	−29.0 ± 12.4	−22.00 ± 0.05	0.53 ± 0.03
Ag HEC	29.0 ± 4.0	25.0 ± 0.4	0.86	+4.4 ± 4.3	+0.06 ± 0.05	0.01 ± 0.02

### 2.5. Nanoparticle tracking analysis

The average hydrodynamic radius of Ag NPs in water was characterized using nanoparticle tracking analysis (NTA) developed by Malvern Instruments Limited (Wiltshire, UK). This technique uses the properties of light scattering and Brownian motion to obtain particle size distributions of samples in liquid suspension [31]. A NS500 instrument, equipped with a 405 nm laser in conjunction with software version NTA 3.1, was used for the purpose of this study. Ag NPs at the concentration of 200 µg ml^−1^ in Milli-Q water were vortexed for 5 s to disperse the particles and then diluted at 0.2 µg ml^−1^. The four different dispersions were then analysed via NTA for the measurement of hydrodynamic diameter at room temperature. All measurements were carried out three times in Milli-Q water to match the *R*_g_ determination. Results are reported as average mode ± standard deviation.

### 2.6. Zeta potential

Zeta potential of Ag NPs (200 µg ml^−1^), diluted 10-fold in Milli-Q water and Dulbecco's Modified Eagle Medium (DMEM), were evaluated using a Zetasizer Nano Z (ZEN5600, Malvern Instruments, UK). Three zeta potential measurements were taken for each sample, each made of 20 accumulations. Measurements were carried out at 25°C, and elaborated using a Smoluchowski model.

### 2.7. Transmission electron microscopy

A Jeol 2100 transmission electron microscope (TEM; USA) was used to image the Ag NPs, with sizes of the Ag NPs being calculated using Image J software. A droplet of each preparation was deposited on a glass slide and left for 30 min. Then a grid was dragged onto the droplet surface to collect the nanoparticles, which accumulate on the droplet surface because of surface tension. Each grid was left to dry and then analysed.

### 2.8. Incubation with the silver nanoparticles

Ag NPs dispersed in a stock solution at a concentration of 200 µg ml^−1^ in Milli-Q water were diluted in the following way: for the cell treatments they were diluted in medium to reach the desired range of concentrations (2.5–100 µg ml^−1^), whereas for the antibacterial activity they were diluted in Milli-Q water to reach a range of concentrations from 0.625 to 100 µg ml^−1^.

### 2.9. Antibacterial activity testing

The antibacterial effect of the nanoparticles was tested against *Escherichia coli*. *Escherichia coli* strain TOP10 or CFT073 harbouring plasmid pGen-Lux [32] were incubated with various concentrations of nanoparticles over a time course of up to seven days. Plasmid pGen-Lux encodes the *lux* operon from Photorhabdus, the gene products of this operon imparts bioluminescence specifically on viable bacteria. One relative light unit (RLU) is approximately equal to 100 viable bacteria. Dead or non-viable bacteria are non-luminescent. All nanoparticles were tested up to a concentration of 100 µg ml^−1^ and were found to be non-luminescent, thus the effects of the particles on bacterial viability were amenable to measurement by luminometry. Bacteria were cultured at 37°C with shaking at 200 r.p.m. in L broth (Sigma, St Louis, MO, USA) to mid-logarithmic phase. Fifty microlitre aliquots of mid-logarithmic cultures (equivalent to approx. 10^6^ cells) were incubated with an equal volume of the requisite nanoparticles in Lumitrac 200 96-well plates (Greiner). Milli-Q water was used as a negative control. Luminescence was read in a Thermofisher Luminoskan™ ascent microplate luminometer (Dublin, Ireland). Each concentration was tested in duplicate, and the experiment was repeated three times. The RLUs of untreated samples were normalized to 100% and treated samples were adjusted accordingly.

### 2.10. Cell culture and experimental treatments

The viability tests after exposure to Ag NP preparations were performed onto A431 (human epidermoid carcinoma) and HaCaT (human keratinocytes) cell lines representative of human skin models. A431 cells were obtained from ATCC (LGC Standard, UK) and cultured in Dulbecco's modified Eagle's medium (DMEM High Glucose) supplemented with 10% FBS, 2 mM L-glutamine, streptomycin (100 µg ml^−1^) and penicillin (100 U ml^−1^). HaCaT cells, obtained from ATCC (LGC Standard, UK), were cultured in DMEM (Dulbecco's modified Eagle's medium with Low Glucose) supplemented with 10% FBS, 2 mM L-glutamine, streptomycin (0.01 µg ml^−1^) and penicillin (0.01 U ml^−1^). Cells were routinely cultured in a humidified atmosphere of 5% CO_2_ in air in T75 cell culture flasks (Nunc, Fisher Scientific, Dublin, Ireland) For cytotoxicity experiments and ELISA assay, cells were seeded in complete growth medium on Nunc-96-well multiwell plates, at a density of 10 × 10^3^cells well^−1^ and 20 × 10^3^cells well^−1^, for A431 and HaCaT, respectively. For the recovery experiments cells were seeded in complete growth medium on Nunc-96-well multiwell plates, at a density of 2 × 10^3^cells well^−1^ and 4 × 10^3^cells well^−1^, for A431 and HaCaT, respectively. After 24 h, the growth medium of the cells was replaced with Ag NPs prepared as previously described. FBS was not employed for this stage to avoid the formation of artefact through protein corona effects. Doses of NPs were adjusted so as to obtain a silver concentration range from 4 µg cm^−2^ to 160 µg cm^−2^ (corresponding to a range from 2.5 to 100 µg ml^−1^). For the particle specific activity, the doses used were adjusted to obtain a final silver concentration of 5, 10, 20 µg ml^−1^ for Ag Pristine, 5, 10, 15 µg ml^−1^ for Ag PVP and 4, 6, 8 µg ml^−1^ for Ag HEC, corresponding to 3.12, 6.24, 12.48 µg cm^−2^, 3.12, 6.24, 9.36 µg cm^−2^ and 2.5, 3.75 and 5 µg cm^−2^ respectively. Since the fractionation led to dilution of the preparation, lower concentrations of total silver were used to assess toxicity. After 24 h exposure cell viability was assessed. In all the experiments, vehicle (1 : 1, Milli-Q water: DMEM) was added as negative control.

### 2.11. Calcein assay

Live cells are distinguished by the presence of ubiquitous intracellular esterase activity, determined by the enzymatic conversion of the virtually nonfluorescent cell-permeant calcein AM to the intensely fluorescent calcein. The polyanionic dye calcein is well retained within live cells, producing an intense uniform green fluorescence in live cells. After 24 h of incubation in the presence of Ag NPs, cell viability was tested replacing medium with a solution of calcein (1 mM) in serum-free medium. After 45 min of incubation at room temperature, protected from light, fluorescence was read at 635 nm with an Epoch microplate reader (Epoch, BioTek, UK). Since nanomaterials could interfere with this assay, a preliminary experiment was performed incubating both dyes with Ag NP preparations at the highest concentration used (100 µg ml^−1^). No fluorescence signal was detected above the background signal.

### 2.12. Resazurin assay

Resazurin is a substrate that changes colour in response to metabolic activity. It is a nonfluorescent molecule converted by intracellular enzymes in the fluorescent compound resorufin (*λ*_em_ = 590 nm). After 24 h of incubation in the presence of Ag NPs, cell viability was tested replacing medium with a solution of resazurin (44 mΜ) in serum-free medium. After 1 h of incubation, fluorescence was measured at 604 nm with Epoch microplate reader. Also in this case we performed a preliminary experiment to test the interference of Ag NPs with resazurin assay. No fluorescence signal was detected above the background.

### 2.13. Lactate dehydrogenase cytotoxicity assay

Lactate dehydrogenase (LDH) is a cytosolic enzyme present in many different cell types. Plasma membrane damage releases LDH into the cell culture media. Extracellular LHD in the media can be quantified with Pierce LDH cytotoxicity assay kit (Thermo Scientific, UK). LDH catalyses the conversion of lactate to pyruvate via NAD+ reduction to NADH. Diaphorase then uses NADH to reduce the tetrazolium salt (INT) to a red formazan product that can be measured at 490 nm. The level of formazan formation is directly proportional to the amount of LDH released into the medium, which is indicative of cytotoxicity. In summary, after 24 h of incubation with Ag NPs, 50 µl of medium were transferred to a 96-well plate. Then, 50 µl of reaction mixture was added to each sample and, after 30 min of incubation at room temperature, 50 µl of stop solution was added. The absorbance was read at 490 and at 680 nm. To determinate the LDH activity, the value of absorbance at 680 nm (background) was subtracted from the 490 nm absorbance before calculation of per cent cytotoxicity. Total LDH activity (maximum LDH release control activity) was used as positive control and was performed by adding 10× lysis buffer (contained in the kit) to the cells.

### 2.14. Cytokine secretion

Details of the Materials, Methods and Results of the Cytokine secretion section are available in the electronic supplementary material.

### 2.15. Statistics

Statistic evaluation of effects has been performed with one-way ANOVA with a Bonferroni test. Statistics have been performed using GraphPad Prism™ software version 4.00 (GraphPad Software Inc., San Diego, CA). Differences have been considered significant for values of *p* < 0.05.

## 3. Results

### 3.1. Physicochemical properties of nanoparticles: size, shape, surface charge and ionic content

**Figure 1.**
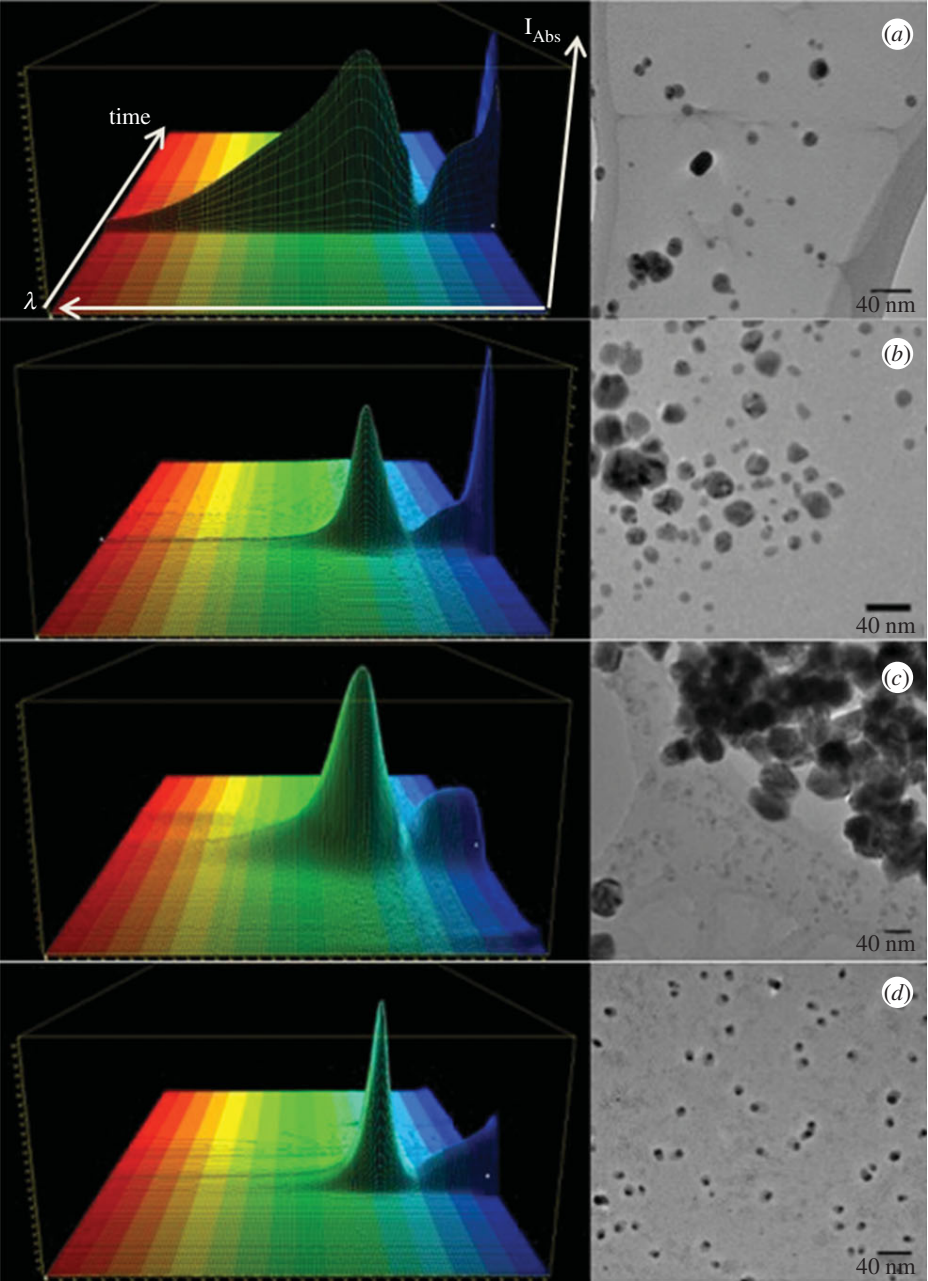
3D absorption spectra acquired in flow during HF5 characterization and NPs collection and representative TEM images of Ag Pristine (*a*), Ag PVP (*b*), AG CIT (*c*) and Ag HEC (*d*). Scale bar of TEM images represents 40 nm. For 3D spectra, horizontal axis represents wavelength (nm), depth axis represents time (min), height axis represent absorption intensity (mAU). Ag Pristine: min: 330 nm, max 450 nm. Ag PVP: min 325 nm, max 425 nm. Ag CIT: min 327 nm, max 420 nm. Ag HEC: min 320, max 410 nm.

Four different Ag NPs have been tested to correlate the particles antiseptic activity and toxicity to their physicochemical properties: Ag Pristine (commercial sample provided by Colorobbia SpA), Ag PVP, Ag CIT and Ag HEC, (CNR-ISTEC synthesized samples), respectively, coated by polyvynilpyrrolidone, citrate and hydroxyethilcellulose added during sol–gel synthesis as stabilizers. These preparations were characterized in terms of size, shape, charge and ionic content (table 2). By using a soft fractionation technique, the HF5 coupled with MALS, we exploited a hyphenated analytical platform able to in-flow size-separate analyte while calculating the gyration radius (*R*_g_) of the particles, which—correlated to the hydrodynamic one (*R_h_*) gives the shape factor. The absorption profile measured online for each preparation is reported in figure 1(*a*(i)), where the three dimensional (3D) absorption spectrum—collected during the separation is shown. With the exclusion of Ag Pristine, all the particles present a sharp absorption peak in a range between 395 and 425 nm, in accordance with the expected plasmon resonance of Ag NPs at such dimensions [33]. The broadening of the Ag Pristine absorption peak towards larger wavelengths can represent the influence of a small population of non-spherical particles. However, it is more likely that the lack of a stabilizing coating caused a partial, although minimal, aggregation process and the forming of aggregates are responsible for a red shift. In fact, we previously observed that PVP-stabilized nanoparticles tend to form chain-shaped aggregates when destabilized [30]. It is interesting to note that while TEM images (figure 1) are included as a comparison, the associated HF5-MALS fractograms (see the electronic supplementary material, figure S2) show a mono-modal size distribution, with only one band at each defined retention time. Furthermore, TEM images show a variety of species of different size and shape. Indeed, while drying Ag NPs could rearrange, agglomerate and nucleate into platelets, and cannot reflect the original state of the sample. Additionally, big agglomerates are usually omitted in TEM analysis. It is also difficult to assess aggregation by TEM owing to drying artefacts that can result in NP agglomeration during sample preparation [34]. Hence, all the calculations and the predictions have been made basing onto size/shape assessment performed in suspension. Hydrodynamic radius measurement, performed with NTA, was determined in Milli-Q water to match MALS measurements, in order to estimate particles shape [31]. Compared to dynamic light scattering (DLS), where the analysis is weighted towards larger particle size, and, therefore, tends to overestimate them [35], NTA has a lower concentration detection limit, and analyses NPs on a particle-by-particle basis. Among the four preparations, Ag PVP particles were the smallest whereas the other three were of similar dimensions (table 2). After the determination of the different radii, we considered the shape factor. This simple comparison of measurable dimensional parameters can in fact provide valuable information about the conformation/shape of particles in solution [36]. Observing the reported *R*_g_/*R*_h_ ratios (table 2), ranging from 0.49 to 0.86, the particles appear to be spherical with a solid core and a less dense coating. In particular, even though the synthesis method is the same for Ag Pristine and PVP, both the difference in absorption spectra and *R*_g_/*R*_h_ forecast a different behaviour between the two. Pristine nanoparticles have a smaller core compared to the coating, which is wider than that of Ag PVP particles, as confirmed by the low *R*_g_/*R*_h_ ratio; indeed even commercial metal nanosols are stabilized by high amount of organic capping agents. Ag PVP, Ag CIT and Ag HEC have a spherical and coated shape as well, whereas Ag HEC shows a very compact nature and, therefore, a very thin coating. The zeta potential of the Ag PVP particles was found to be negative in pure water owing to the interaction of the surface with gluconate residue (generated during synthesis), but it was at least partially neutralized in medium (table 2). Ag Pristine particles undergo the same effect even though the decrease is less drastic. Ag CIT particles showed the expected negative charge, while Ag HEC had a neutral/weakly positive potential. The lower absolute values of zeta potential measured in medium, predict a low electrostatic repulsion even though from a visual observation of dispersed samples, the steric component of the coating seems to prevent the solid coagulation, segregation and sedimentation. In fact, a layer of ligands creates a repulsive potential to counteract the attractive van der Waals force [37]. This repulsion can be of steric nature (coating with polymers, such as PVP or uncharged molecules) or of electrostatic nature (coating with charged ligands, such as citrate). One direct consequence is that an increase in the ionic strength of the solution will shield the electrostatic repulsive potential, and lead to the aggregation of the nanoparticles, or their heterocoagulation onto living membranes [38]. The ionic content of Ag Pristine and Ag PVP is similar to predictable, because they were obtained through similar synthesis routes. Differences in activity and toxicity then can be dependent on the nature of the particles, in particular we expected Ag Pristine to be more active and toxic because the hard silver-based core is smaller. Citrate and HEC-coated particles have a considerably lower amount of free ions in the solution owing to the more precise stoichiometry applied to the synthesis (table 2). Being that the ionic percentage of Ag CIT particles is higher by an order of magnitude compared to Ag HEC, a consequent higher activity would have been expected if one considers the ionic bioavailability as the only mechanism of action. Nevertheless, the results we obtained showed an opposite trend, which makes necessary taking into account surface charge/particle specific activity. All the Ag NPs tested, are of a spherical shape; hence this parameter is kept constant. However, size, coating, charge and ion release vary for the four preparations and have been evaluated individually and as a combination. In this work, all the physical and chemical characterization of Ag NPs used has been made in suspension (water or cell medium), to allow us to predict the ‘real’ activity of nanoparticles *in vitro* for each preparation and to justify differences in antiseptic/toxic behaviour between similar preparations. In fact, the properties of Ag NPs can change during the life cycle of a nanomaterial and are partly depending on interactions with the surrounding environment, which may lead to a different behaviour of nanomaterials in different situations [39].

### 3.2. Antimicrobial activity of silver nanoparticles over time and under re-contamination

**Figure 2.**
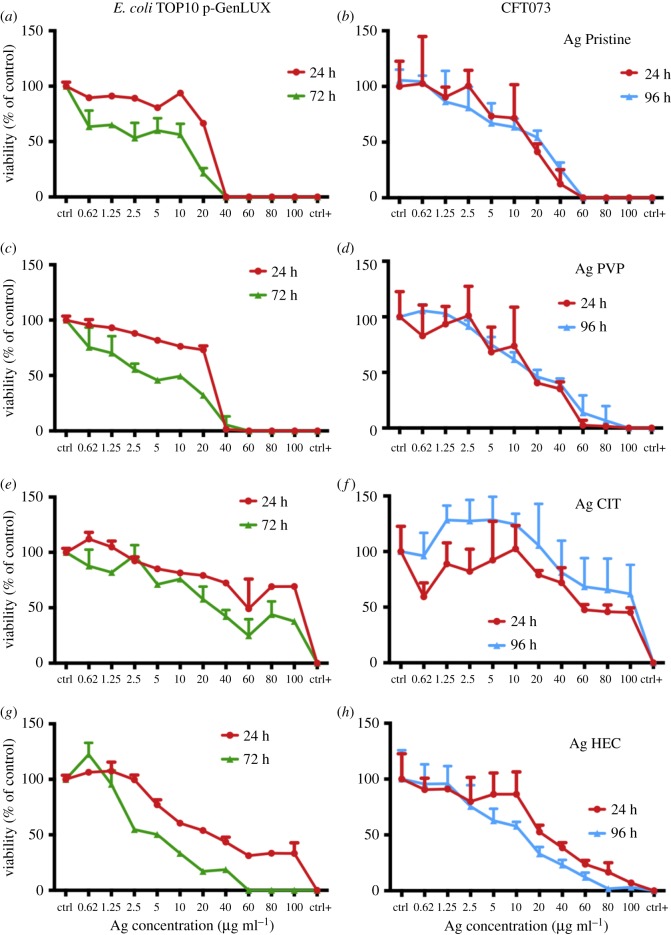
Viability of bacterial strains *E. coli* Top10 and CFT073 after 24 h (red line) and 72 or 96 h (green or blue line). Bacteria were cultured at 37°C with shaking at 200 r.p.m. in L broth to mid-logarithmic phase. Of note, 50 µl aliquots of mid-logarithmic cultures (equivalent to approx. 10^6^ cells) were incubated with an equal volume of Ag NPs (from 0.62 to 100 µg ml^−1^ final concentration) in 96-well plates. Milli-Q water was used as a negative control and ethanol as a positive control. After 24, 72 and 96 h luminescence was read and the RLUs of untreated samples were normalized to 100% (see Methods). (*a,c,e,g*) *Escherichia coli* TOP10; (*b,d,f,h*) *E. coli* CFT073. Data are means of three independent determinations ± s.d. Ag Pristine (*a,b*) Ag PVP (*c,d*) Ag CIT (*e,f*) and Ag HEC (*g,h*).

**Figure 3.**
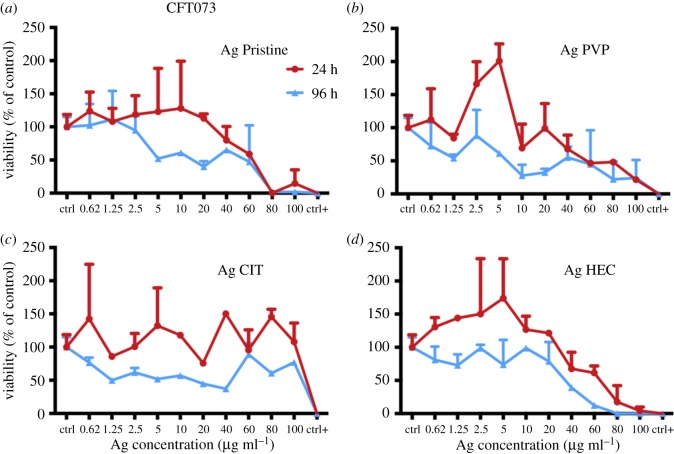
Viability of bacterial strain CFT073 after 24 h (red line) and 96 h (blue line) against reused Ag NPs. Bacteria were cultured at 37°C with shaking at 200 r.p.m. in L broth to mid-logarithmic phase. Of note, 50 µl aliquots of mid-logarithmic cultures (equivalent to approx. 10^6^ cells) were incubated with an equal volume of water in the 96-well plates containing the previously employed nanoparticles at different concentrations (from 0.62 to 100 µg ml^−1^ final concentration). The same well employed for the previous experiment was used as a negative control while ethanol was added instead of water as a positive control. After 24 and 96 h luminescence was read and the RLUs of untreated samples were normalized to 100% (see Material and methods). (*a*–*d*): CFT073. Data are means of three independent determinations ± s.d. Ag Pristine (*a*), Ag PVP (*b*), Ag CIT (*c*) and Ag HEC (*d*).

We set different time points for bactericidal activity to simulate ageing of the medical device. Then, we designed a way to test the ability of Ag NPs to display a long lasting antiseptic effect. Lastly, the purified nanoparticles obtained through HF5 were tested to identify particle-specific effects. An important step is the choice of an appropriate model. *Escherichia coli* is a good model to test antibacterial activity of nanoparticles [40], however it is prudent to measure the activity of nanoparticles against pathogenic strains, because these are more likely encountered in a clinical situation. Bacterial strains TOP10 (a K-12 isolate) and CFT073 were chosen for analysis. Strain CFT073 is a uropathogenic strain that can form biofilms and cause urinary tract infections [41]. It belongs to sequence type 73 (ST73) of pathogenic *E. coli* and is one of the most frequent causes of *E. coli* extraintestinal infection. To maximize the information obtainable, the luminescence reads were performed until the negative control displayed a decrease in intensity when compared to the initial value. In this way, every experiment is balanced on the strain tested and the effect of Ag NPs can be evaluated over the longest period possible, accounting for strain-to-strain variability. *Escherichia coli* TOP10 was incubated with the nanoparticles over a time course of up to 72 h, while CFT073 allowed us to carry on the experiments up to 96 h without significant loss of luminescence on the untreated control (figure 2). At 24 h of exposure (red lines) the *E. coli* was not viable at concentrations ≥40 µg ml^−1^ for Ag Pristine and Ag PVP (figure 2*a*–*c*), while Ag CIT and Ag HEC displayed a smaller effect. Indeed, at the concentration of 100 µg ml^−1^ of Ag HEC viability dropped to 30% (figure 2*g*). After 72 h of exposure, a similar trend was registered for Ag Pristine and Ag PVP, with a decrease of viability to 75% and 65%, for Ag Pristine and Ag PVP respectively, even at the lowest concentration (figure 2*a*–*c*). However, Ag HEC induced a clear-cut killing effect for *E. coli,* with a complete loss of viability at concentrations ≥60 µg ml^−1^. On the contrary, Ag CIT was the least effective, with a decrease of viability to 50% for the highest concentration used (figure 2*e*). The particles were then tested against the clinical isolate CFT073 and all these agents displayed a bactericidal effect. Comparing these results to those obtained with *E. coli* TOP10 we observed a reduced sensitivity to all the preparations. CFT073 showed a higher resistance to Ag Pristine and Ag PVP, and the complete loss of viability was only observed at a concentration of ≥60 µg ml^−1^ (figure 2*b*–*d*), whereas Ag CIT displayed a mediocre effect (figure 2*f*). None of the three preparations showed a time-dependent antimicrobial effect. Instead, Ag HEC showed a time and dose dependent toxicity, with a complete loss of viability at doses ≥80 µg ml^−1^, thus maintaining its activity even towards a more resilient strain. This observation is in contrast with what could be expected when only applying the direct relationship between free ions and toxicant activity, which confirmed the presence of particle-specific interaction with living organisms. To test the ability of Ag NPs to maintain their antiseptic effect, an alternative experimental strategy was used. The used plates from the previous experiment with CFT073 were left to dry, the lack of remaining living bacteria was confirmed and another inoculation of viable luminescent bacteria was performed (figure 3). CFT073 viability was affected in a time-dependent manner when bacteria were treated with Ag NPs that had previously been used to test killing. The overall antiseptic effect was lower in comparison to that of freshly prepared nanoparticles, and one of the concurring factors is that particles were not uniformly distributed in the wells, because they were only left to dry after the previous experiment. After 24 h, particles are ineffective (Ag CIT) or only effective for the highest concentrations: this lack of acute toxicity (when compared to the previous experiment) finds explanation in the lack of starting free Ag^+^. This is also confirmed by the fact that Ag HEC displayed an almost unvaried dose–response pattern (figures 2*h* and 3*d*). All the preparations except for Ag CIT caused a time-dependent decrease in bacterial viability, supporting the hypothesis of the particles being interacting with bacteria.

### 3.3. Cytotoxicity assessment of silver nanoparticles with two relevant cell models: A431 and HaCaT cells

**Figure 4.**
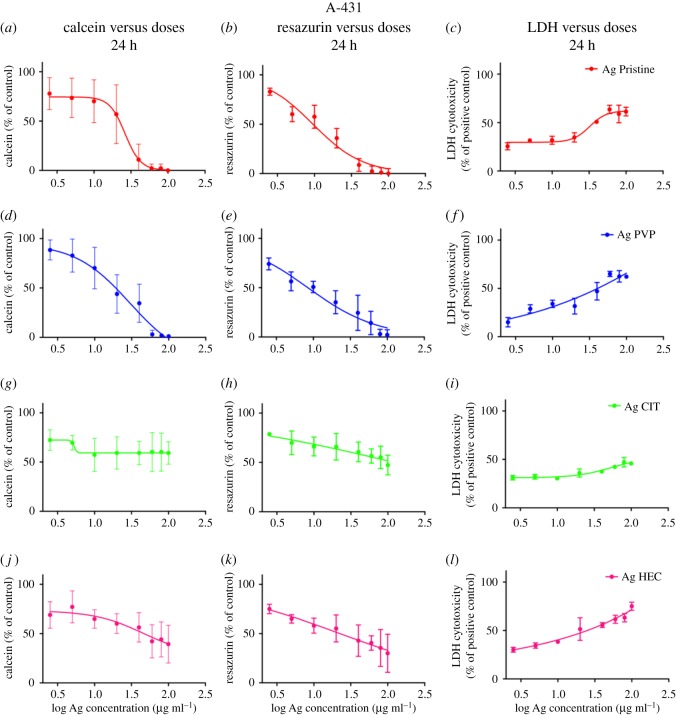
Cytotoxicity of Ag NPs towards A431 cell line. Cells, grown for 24 h in complete growth medium, were treated with different concentrations of Ag NPs or with ethanol (80%), used as a positive control. After 24 h of exposure cell viability was assessed using calcein assay, resazurin assay or LDH assay (see Material and methods). (*a,d,g,j*) Calcein assay; (*b,e,h,k*) resazurin assay; (*c,f,i,l*) LDH assay. Data are means of three independent determinations ± s.d. Ag Pristine (*a*–*c*), Ag PVP (*d*–*f*), Ag CIT (*g*–*i*) and Ag HEC (*j*–*l*).

**Figure 5.**
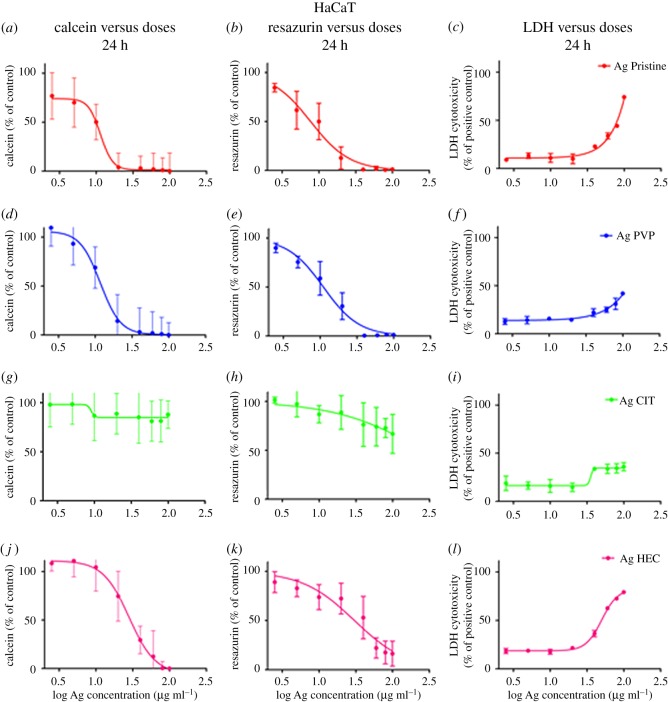
Cytotoxicity of Ag NPs towards HaCaT cells. Cells, grown for 24 h in complete growth medium, were treated with different concentrations of Ag NPs or with ethanol (80%), used as a positive control. After 24 h of exposure cell viability was assessed using calcein assay, or resazurin assay, or LDH assay (see Material and methods). (*a,d,g,j*) Calcein assay; (*b,e,h,k*) resazurin assay; (*c,f,i,l*) LDH assay. Data are means of three independent determinations ± s.d. Ag Pristine (*a*–*c*), Ag PVP (*d*–*f*), Ag CIT (*g*–*i*) and Ag HEC (*j*–*l*).

To determine the safety of the Ag NP candidates for use as medical device we individuated the most relevant exposure scenario according to the SCENIHR guidelines [19]. Their use is meant to be as a surface coating, and can be then defined ‘surface contacting’, it can interact with consumers (workers, patients) through contact thus ‘facing/interacting with skin tissue’, and is not meant for topical use hence the scenario is of ‘limited contact’ (= or less than 24 h) [7]. Investigating the effect of Ag NPs on two human skin models is useful because they represent different skin layers. A431 are representative of the outer skin layer while HaCaT (keratinocytes), also part of the *stratum granulosus* of the skin, can simulate *in vitro* the first effects of penetration of the nanoparticles [29,42–45]. Different mechanisms of cytotoxicity, by carrying out three *in vitro* assays where the toxic effects of Ag NPs towards human skin cells were assessed (figures 4 and 5) in terms of presence of viable cells (calcein assay), damaging of cell membranes (LDH assay), or cellular metabolism (resazurin assay). Moreover, the cytotoxicity results obtained allowed us to calculate the IC_50_ for each preparation of Ag NPs and to compare the values of IC_50_ obtained between the different assays (table 3). A431 and HaCaT cells were exposed to the four preparations of Ag NPs and after 24 h the cell viability was assessed (figures 4 and 5, respectively). A431 and HaCaT cells reacted similarly to a 24 h exposure to Ag Pristine. Indeed this preparation induced an evident decrease in cell viability with calcein assay (figures 4*a* and 5*a*), whereas resazurin assay showed a dose-dependent decrease in viability (figures 4*b* and 5*b*). Moreover, LDH assay showed, for both cell lines, a cytotoxic effect starting from 20 µg ml^−1^, with an increase in LDH activity of 60–70% for the highest dose used (100 µg ml^−1^) (figures 4*c* and 5*c*). Ag PVP induced a similar cytotoxic effect onto A431 and HaCaT cells when compared to Ag Pristine. Both calcein and resazurin assay showed a dose-dependent decrease in cell viability (figures 4*d*,*e* and 5*d*,*e*). LDH assay confirmed the obtained results (figures 4*f* and 5*f*). On the contrary, Ag CIT, in both cell lines, did not show any cytotoxic effect (figure 4*g*–*i* and 5*g*–*i*) and the values of IC_50_ were greater than 100 µg ml^−1^ both for calcein and resazurin assays. Lastly, Ag HEC showed a moderate dose-dependent toxicity in both cell lines (figures 4*j*–*l* and 5*j*–*l*). However, no complete loss of viability was observed even at the highest concentrations used. In regard to the sensitivity of the assays, in our study we observed that calcein assay appears to be less sensitive compared with resazurin in detecting decrease of cell viability upon exposure to Ag NPs. The cytotoxic effect of Ag NPs on skin cell models was also investigated through the quantification of the cytokines TNF-α, IL-6, IL-8 and IL-1*β* secreted upon exposure (electronic supplementary material, figure S3). Ag HEC displayed a lack of acute inflammatory response, which can be a promising feature for surface treating applications. Even though skin contact is a possible pathway for contamination, a long-term effect for human health is avoided.

**Table 3.** IC_50_ values (µg ml^−1^) of A431 and HaCaT cells exposed for 24 h to Ag NPs.

	A431		HaCaT	
IC_50_ values (µg ml^−1^)	calcein	resazurin	calcein	resazurin
Ag Pristine	21.87	9.33	10.00	7.58
Ag PVP	18.62	8.31	12.02	10.96
Ag CIT	>100	>100	>100	>100
Ag HEC	45.7	21.87	28.84	29.51

**Figure 6.**
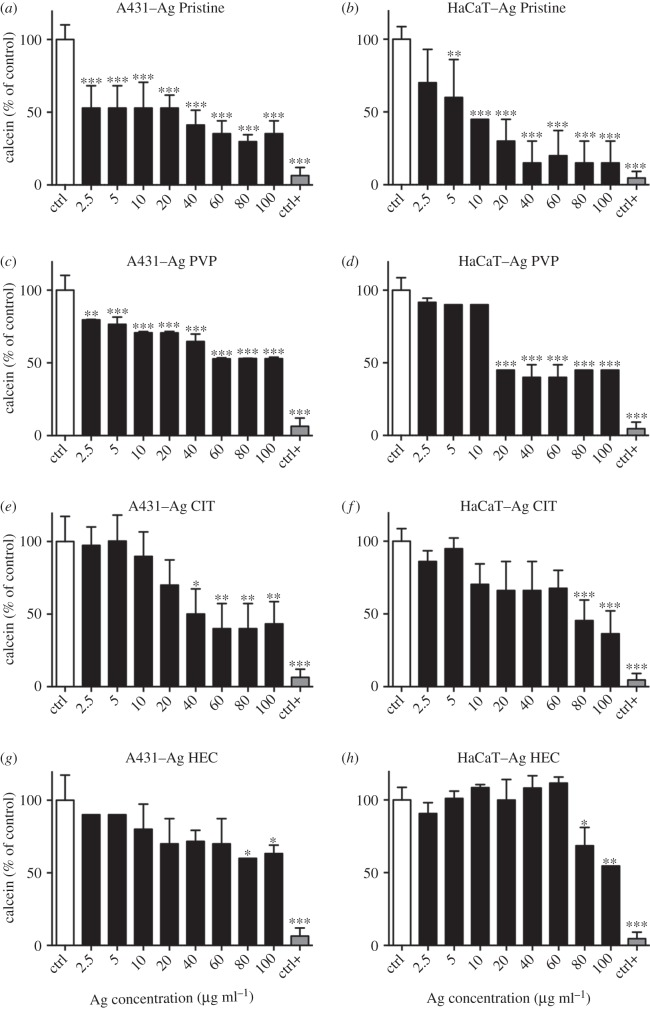
Recovery A431 and HaCaT cells after exposure to Ag NPs. Cells, grown for 24 h in complete growth medium, were treated with different concentrations of Ag NPs or with ethanol (80%), used as a positive control. After 24 h of exposure cell medium was replaced with full growth medium and cells were cultured for six additional days. On the seventh day viability was assessed using calcein assay. (*a,c,e,g*) A431; (*b,d,f,h*) HaCaT. Data are means of three independent determinations ± s.d. *p < 0.05, **p < 0.01 and ***p < 0.001 versus untreated, control cells. Ag Pristine (*a,b*), Ag PVP (*c,d*), Ag CIT (*e,f*) and Ag HEC (*g,h*).

To investigate the effect of long-lasting toxicity of Ag NPs, A431 and HaCaT cells were exposed for 24 h to the four preparations of Ag NPs. Then, the cells were rinsed and allowed to recover in a complete growth medium for additional 6 days (figure 6). After the recovery period of 6 days, an increase in viability (assessed with calcein assay) of both cell lines treated with Ag NPs was observed, especially for Ag Pristine, Ag PVP and Ag HEC at the higher concentrations used (60–80–100 µg ml^−1^), as shown in figure 6. Moreover, the recovery experiment confirmed the lower cytotoxicity of Ag CIT (figure 6*e*,*f*). In summary, the cellular toxicity followed the same pattern we observed for bacterial strains, with a higher toxicity for Ag Pristine and Ag PVP, and a lower and moderate toxicity for Ag CIT and Ag HEC, respectively.

### 3.4. Specific toxicity and antimicrobial activity of flow-field flow fractionated silver nanoparticles

**Figure 7.**
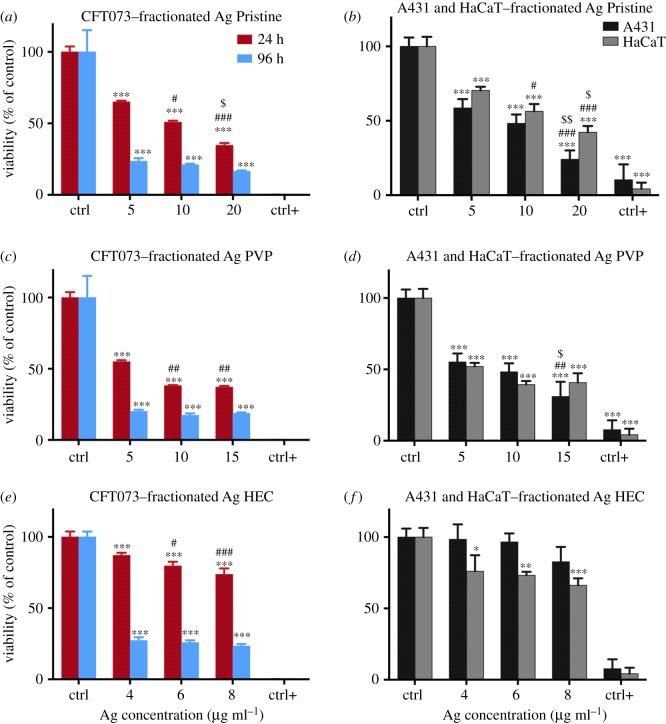
Viability of bacterial strain CFT073 after 24 and 96 h (*a,c,e*) and skin cells (*b,d,f*) after 24 h when treated with fractionated Ag NPs. (*a*–*e*) Bacteria were cultured at 37°C with shaking at 200 r.p.m. in L broth to mid-logarithmic phase. Of note, 50 µl aliquots of mid-logarithmic cultures (equivalent to approx. 10^6^ cells) were incubated with an equal volume of Ag NPs (5, 10, 20 µg ml^−1^ for Ag Pristine, 5, 10, 15 µg ml^−1^ for Ag PVP and 4, 6, 8 µg ml^−1^ for Ag HEC) in 96-well plates. Milli-Q water was used as a negative control and ethanol as a positive control. After 24 and 96 h luminescence was read and the RLUs of untreated samples were normalized to 100% (see Material and methods). (*a,c,e*): CFT073. Data are means of three independent determinations ± s.d. *p < 0.05, **p < 0.01 and ***p < 0.001 versus untreated, control bacterial cells. # *p *< 0.05 and ## *p *< 0.01 versus 5 µg ml^−1^ Ag Pristine; ; $ *p *< 0.05 versus 10 µg ml^−1^ Ag Pristine;: ## *p *< 0.01 versus 5 µg ml^−1^ Ag PVP; : # *p *< 0.05 and ### *p *< 0.001 versus 4 µg ml^−1^ Ag HEC; versus 4 µg ml^−1^. (*b,d,f*) Cells, A431 and HaCaT, grown in complete growth medium for 24 h, were treated with different concentrations of Ag NPs (5, 10, 20 µg ml^−1^ for Ag Pristine, 5, 10, 15 µg ml^−1^ for Ag PVP and 4, 6, 8 µg ml^−1^ for Ag HEC) or with ethanol (80%) used as a positive control. After 24 h of exposure cell viability was assessed using calcein assay. Data are means of three independent determinations ± s.d. *p < 0.05, **p < 0.01 and ***p < 0.001 versus untreated, control bacterial cells. # *p *< 0.05 and ### *p *< 0.001 versus 5 µg ml^−1^ Ag Pristine; $ *p *< 0.05 and $$ *p *< 0.01 versus 10 µg ml^−1^ Ag Pristine; ## *p *< 0.01 versus 5 µg ml^−1^ Ag PVP; $ *p *< 0.05 versus 10 µg ml^−1^ Ag PVP. Ag Pristine (*a,b*), Ag PVP (*c*,*d*) and Ag HEC (*e*,*f*).

In order to assess the toxic and antiseptic effects ascribable to nanoparticles themselves, isolated from the suspension of each Ag NP sample and from the starting concentration of Ag^+^, we treated both bacteria and cell lines with the FlFFF fractionated nanoparticles, according to the FlFFF process described in the Material and methods section. The concentrations used for the specific exposure are calculated, keeping in consideration the dilution factor occurred during fractionation and the measured initial Ag^+^ amount. These are 5, 10, 20 µg ml^−1^ for Ag Pristine, 5, 10, 15 µg ml^−1^ for Ag PVP and 4, 6, 8 µg ml^−1^ for Ag HEC. On these, viability of bacterial strain CFT073 after 24 and 96 h and cytotoxicity has been measured for the three relevant AgNPs and results are shown in figure 7. Of note, citrate-coated particles have not been screened in this further study because their antimicrobial effect was found to be negligible (as shown in figures 2 and 3). Interestingly, Pristine and PVP-coated nanoparticles exerted a similar effect on the pathogenic strain CFT073, at both time points. The antimicrobial activity is preserved and increased with respect to unfractionated samples (e.g. for fractionated Ag PVP we observed a loss of viability of 80% at 15 µg ml^−1^ as opposed to 60% for the unfractionated sample). The acute effect of these two preparations could reflect the fact that nanoparticles establish a new equilibrium by releasing ions in the new medium, and, therefore, a certain (and equal) amount of Ag+ is present both for Ag Pristine and Ag PVP. Ag HEC nanoparticles did not show a strong antimicrobial effect after 24 h, which is linked to their initial lesser amount of free Ag+ ions; we however observed a dose-dependent response. Nevertheless, after 96 h, viability of CFT073 was reduced to 27% even at the very low concentrations used (4, 6, 8 µg ml^−1^) (figure 7*e*). Fractionated Ag Pristine and Ag PVP determined a higher decrease in viability of A431 and HaCaT cells than the unfractionated samples (figure 7*b*–*d*). Ag HEC did not produce any significant toxic effect in A431 cells, and HaCaT viability remained well over 50% when compared to the untreated cells (figure 7*f*).

## 4. Discussion

**Figure 8.**
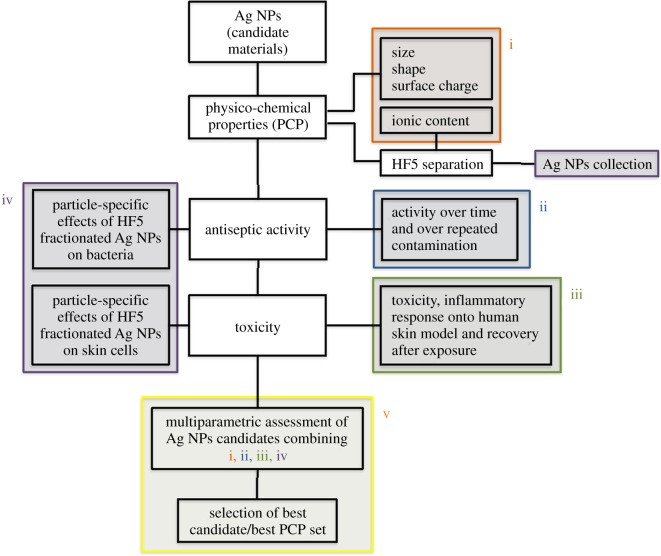
Schematic representation of the multi-step approach used. (i) Characterization of the particles in suspension to match *in vitro* tests, (ii) testing of the nanoparticles to quantify their antibacterial response (acute and in a life-cycle scenario), (iii) *in vitro* test to assess toxic response upon contact (skin model), (iv) testing of collected, purified nanoparticle to assess particle-specific activity, and (v) correlation of relevant properties and nanoparticles activity (antiseptic/toxic and particle-specific).

**Figure 9.**
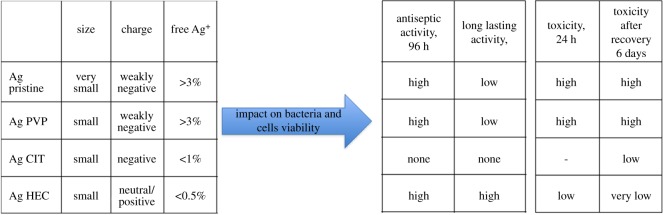
Summary of physico-chemical properties of the four Ag NPs preparations and their impact on skin toxicity and antiseptic activity.

**Figure 10.**
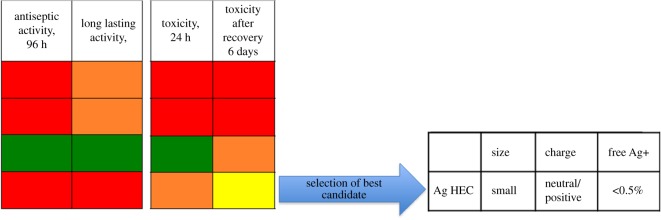
Qualitative representation of positive outcomes and correlated physico-chemical properties of the four candidates. Colours refer to toxicity towards human skin cells/bacteria. Red, high toxicity; orange, low toxicity; yellow, very low toxicity and green, non-toxic.

Our work intends to address the lack of existing platforms to obtain coherent information pertaining the Ag NP samples, and to provide more than simple characterization. In fact, we choose to characterize Ag NPs based on a separation step (achieved with FlFFF) followed by characterization through light scattering techniques and fraction collection, to assess together the antibacterial activity and the toxic response of human cells to different Ag NPs. HF5 is a miniaturized and disposable field-flow fractionation device, with the advantage of a lower channel volume, flow rates and, therefore, lower dilution factors. Both narrow peaks and low dilution contribute to increase detectability and sensitivity [46,47]. Short analysis time and high throughput, ease of use and minimum downtime are determining factors for a productive analytical tool; moreover the sample is analysed in suspension thus not creating artefacts through handling (e.g. drying). The advantage of using characterization techniques that can process samples in suspension, e.g. NTA together with FlFFF, UV–vis detector and MALS, is that realistic size and shape can be determined while the orthogonal data provides the information on the particles' activity [48]. By using a DAD as a concentration detector we were also able to acquire the absorption spectra of the nanoparticles, thus providing a contemporary surface evaluation in terms of different coatings. In fact, when acquired during a separation, an absorption spectrum consists of a 3D plot, where one dimension is time, one is wavelength and the third is intensity of absorption. Last, by coupling graphite FAAS to quantify the collected free ions, a simultaneous quantification of free Ag^+^ and nanoparticles characterization and collection allowed for a direct calculation of ‘nano’ silver, to understand the amount of nano-dimensioned metal with respect to the total [49]. Indeed another advantage of using a miniaturized device such as HF5, is that the collected cross-flow resulted in being concentrated enough to allow for low-cost techniques such as graphite FAAS to be employed, as opposed to ICP-MS. The nature of the separation, and the simultaneous quantification of free ions, allowed for the recollection of separated Ag NPs that could be selectively tested for toxicity and activity. This is a new feature that overcomes the *a priori* calculation of the contributions of ions, medium, and other contaminants and provides a direct quantification of particle-specific effect. In this setting, our approach for the evaluation of Ag NPs was based on a five-step procedure (figure 8), able to accomplish: (i) characterization of the particles in suspension to match *in vitro* tests, (ii) testing of the nanoparticles to quantify their antibacterial response (acute and in a life cycle scenario), (iii) *in vitro* test to assess toxic response upon contact (skin model), (iv) testing of collected, purified nanoparticles to assess particle-specific activity, and (v) correlation of relevant properties and nanoparticles activity (antiseptic/toxic). The ranking and evaluation of the most suitable Ag NPs across the four under investigation to be used as an antimicrobial agent with reduced acute toxicity was performed by taking into account all the parameters involved from the physicochemical properties (PCP) to the *in vitro* and antiseptic response. Our results reported a highest toxicity (assessed with viability assays and the determination of cytokines secretion) towards human skin cells and antiseptic activity on bacteria, respectively, for both Ag Pristine and Ag PVP, suggesting that the higher content of Ag^+^ plays a crucial role in determining the toxicity of Ag NPs (figures 2–5) [50]. However, the effect on the viability of CFT073, A431 and HaCaT cells was evaluated also using the fractionated and collected nanoparticles where the only available silver was the solid phase contained in the nanostructure and the ionic phase adsorbed on it. The results showed a higher toxic/antiseptic effect compared to that of the entire sol (figure 7). These results seem to correlate with the hypothesis of a particle size-dependent activity/toxicity. Particle size plays an important role in toxicity and antimicrobial activity; many studies suggest that smaller particles have a higher chance to interact with the cell membrane and are, therefore, more toxic [11,51,52]. A direct prediction and comparison in activity (versus bacteria) and toxicity (versus human cell lines) is possible between Ag Pristine and Ag PVP, where size was the main distinguishing factor, as reported in table 2. Ag Pristine and Ag PVP derived from similar sol–gel synthesis route [25] were obtained with different reagents concentration and this led to both different dimension and a different absorption spectrum. We predicted that the different *R*_g_/*R*_h_ ratio—hence the different coating thickness---together with the bigger size, could lead to a lesser activity of Ag Pristine, because a lower ratio is indicative of a thicker layer of polymer onto the surface of the particles, making the active surface less available for interactions. Nevertheless, the silver ion content, which has been quantified through flow field flow fractionation and atomic absorption, is almost the same between the two preparations (table 2). Fractionated Ag PVP, as showed in figure 7*a*–*c*, is more active/toxic than fractionated Ag Pristine, and this is coherent with the lower size of Ag PVP particles. In fact, free silver ions can be sequestrated by the medium and made less available, while the particles—being stable—maintain their potential. Moreover, the increase observed for fractionated samples of Ag Pristine and Ag PVP can also be partially owing to a destabilizing effect caused by dilution and subsequent different equilibrium achieved between Ag NPs and free ions, compared to the initial preparations. However, when diluted to obtain stock concentration for the previous experiments the nanoparticles did not show this deviation from linearity, and the dilution effect can be taken out from the concurring parameters to cause destabilization and hence increased toxicity. Purification of nanoparticles through FlFFF is also able to remove the impurities without affecting the characteristics of the nanoparticles. Those impurities present in Ag NPs suspensions do not only include Ag+ but also the residual reducing and stabilizing agents from the synthesis process that could hinder or modify the overall activity. Ag CIT and Ag HEC were expected to be both less toxic and less antiseptic, because the acute effect mediated by ion release is minor. Indeed, they are both less toxic than Ag Pristine and Ag PVP, either to bacteria strains or to the cell lines. For Ag HEC the difference between the activity of unfractionated and fractionated samples was very low (figures 2*g*,*h*, 3*d*, 4*j*–*l*, 5*j*–*l*, 7*e*,*f*), and this can be explained by the fact that the free ion concentration in the starting material was low enough not to interfere particularly with the particle-specific activity. Moreover, our work reported that Ag HEC is more effective in terms of activity and toxicity (viability assays) than Ag CIT, suggesting that other parameters than ionic concentration become relevant in this case. By simply following the theory of silver ions being the active element, Ag CIT should have been toxic (even though less than Ag Pristine and Ag PVP) and Ag HEC should have had a negligible activity, because the difference in ionic content of the two sols is more than one order of magnitude (table 2). However, these two preparations have the same size but we reported opposite surface charge (Ag CIT is negatively charged and Ag HEC is neutral/weakly positive). When considering surface charge of the nanoparticles, one should also consider the counterpart's one, and both cells and bacteria have a negatively charged membrane. Similarly charged particles tend to repulse each other in proportion to the magnitude of the (negative) potential. Therefore, by taking in account surface charge then the lowest activity found in Ag CIT finds explanation and suggests that for a low ionic content (less than 1%), the main role is played by attractive forces [14,50]. As found in previous studies [14], on differently charged particles, as the absolute value of the negative potential decreases, the electrostatic barrier between membranes is reduced and the chance of cell-particle interaction increases, determining a higher toxicity. Repulsion turns to attraction when cells and bacteria are exposed to more positively charged particles like Ag HEC. Hence, when screening for the best Ag NP candidate, particle activity is a relevant aspect to be considered: differently charged materials exert a relevant influence on the overall activity, especially when longer exposure is considered. Therefore, particles cannot be considered only as Ag ion-release devices. When focusing on a real-life scenario, a further step is necessary and it involves accounting for the time of action of the candidate compounds. Indeed, if the 24 h exposure is sufficient to describe skin contact, the antimicrobial potential needs to be monitored over a longer amount of time, and a specific experimental design has to be set up for the scope. In fact, nanoparticles can have a long-term effect and measuring their activity over too short a span can lead to a biased evaluation. This long-term activity is also linked to the fact that bacteria can be selected through the previous use of silver ion-based antiseptics, widely used, that can increase resistance and cancel the acute toxicity determined by free ions [12]. On the other hand, nanoparticles need time to exert their particle-specific interaction and could have a delayed bacterial toxicity. By re-infusing living bacteria on dried plates containing used nanoparticles, the long-lasting antiseptic potential of Ag Pristine, Ag PVP, Ag CIT and Ag HEC was confirmed and so was the particle-specific activity: the dose-dependent effect is similar to that of newly diluted nanoparticles and ion-dependent effect is to be considered negligible (figure 4). The applicability of these nanoparticles, or of similar engineered ones, is desirable as they have shown to be good candidates for surface treatment when the correct time points were considered [53]. In fact the lack of individuation of long-term antiseptic effect would have disqualified Ag HEC, which instead seems to be the most interesting candidate because we did not report an inflammatory effect on human skin cells and, moreover, after the recovery experiment cell viability is above 50% for all the concentration used (figure 6). To summarize the results in a compact view we rationalized the property-effect relationships between the evaluated parameters, establishing a basis to categorize the key parameters needed to predict nanoparticle activity. Figure 9 shows how the different PCP of the Ag NPs can impact toxicity on skin cells and antiseptic activity. By exploiting this multi-step approach, based on characterization, toxicity assessment and activity evaluation, it is possible to extrapolate which combination of PCP is more effective. This result can be expanded over the four candidates screened and represents a process for the selection of the required PCP set needed for a successful antiseptic medical device. This selection is visualized in figure 10. As shown in figures 9 and 10, small particles with a negative surface charge and a higher ionic content like Ag PVP are acutely toxic and, even though antiseptic, have a small window of concentrations that can be exploited for surface treatment because the two trends go accordingly. Even bigger particles, such as Ag Pristine, that possess the same charge and ion content parameters, display a similar behaviour. Strongly negatively charged particles like Ag CIT do not show remarkable effects and are not good candidates. Instead, the combination of a more positive surface charge, a very low amount of free silver ions, and a size above 20 nm leads to the best candidate, represented in this work by Ag HEC. In fact, this preparation showed a low acute toxicity, a good cellular recovery after a 24 h exposure, and a remarkable long-term antiseptic activity. This is especially true for purified particles, where the decrease in bacterial viability was 80% (at 96 h of exposure) even for very low concentrations of nanoparticles.

## 5. Conclusion

In our work, we developed and performed a five-step approach to assess and identify the purpose-specific applicability window of candidate Ag NPs, as antimicrobials in healthcare settings while also protecting consumer safety upon occasional or unintentional exposure.

FlFFF, coupled online with UV and MALS detectors, and offline with atomic absorption, provided together with NTA all the needed information to evaluate each nanoparticle descriptor in a realistic medium, allowing for the determination of the key parameters for the safe development of antiseptic nanoparticles. Moreover, the separation step provided purified particles for individual testing. We evaluated efficacy aspects by monitoring the long-term effect of nanoparticles onto luminescent strains of *E. coli* and of CFT073, a pathogenic strain present in hospitals and responsible for urinary tract infections. We addressed safety aspects by studying toxicity, inflammatory response and cellular recovery upon exposure of skin models to Ag NPs. Lastly, the design of experiments to verify preservation of antiseptic activity and particle-specific effects, led us to a realistic evaluation of the best candidate materials as coating agents, in correlation with their physicochemical requirements.

## Supplementary Material

Supplementary Information

## Supplementary Material

Raw data uploaded on Dryad

## Data accessibility

The datasets supporting the results presented in this article are uploaded as part of the electronic supplementary material in this submission and are available online under the Data Dryad archive (http://dx.doi.org/10.5061/dryad.36j5d) [54] and under the Trinity College Access to Research Archive system (http://hdl.handle.net/2262/81721) under A. P.-M. research publication. This work was also included as part of V.Ms PhD thesis, as a result of her two internships and collaborative work with the co-authors of this manuscript carried out at Trinity College Dublin, Ireland in 2015 and 2016 (supervised by A.P.-M.). (http://dx.doi.org/10.6092/unibo/amsdottorato/7995).
