# Supplementary material for: Silver nanoparticles as a medical device in healthcare settings: a five-step approach for candidate screening of coating agents
Source: R Soc Open Sci. 2018 Jan 31;5(1):171113. doi: 10.1098/rsos.171113 (PMC5792903; doi:10.1098/rsos.171113)
Supplement: Supplementary Information [file rsos171113supp1.docx]

**Silver nanoparticles as a medical device in healthcare settings: a five-step approach for candidate screening of coating agents**

**Supplementary Information**

Valentina Marassi^1^, Luisana Di Cristo^2^, Stephen G. J. Smith^2,3^, Simona Ortelli^4^, Magda Blosi^4^, Anna L. Costa^4^, Pierluigi Reschiglian^1^, Yuri Volkov^2^, Adriele Prina-Mello^2,5*^

^1^ Department of Chemistry "G. Ciamician", Via Selmi 2, 40126 Bologna, Italy

^2^ Department of Clinical Medicine, Trinity Translational Medicine Institute (TTMI), School of Medicine, Trinity College Dublin, Dublin 8, Ireland

^3^ Department of Clinical Microbiology, Sir Patrick Dun Research Laboratory, School of Medicine, Trinity College, Dublin 8, Ireland

^4^ Institute of Science and Technology for Ceramics (CNR-ISTEC), National Research Council of Italy, Via Granarolo 64, 48018 Faenza, RA, Italy

^5^ AMBER Centre and CRANN Institute, Trinity College Dublin, Dublin 2, Ireland

*Corresponding Authors:

**Adriele Prina-Mello, PhD**

School of Medicine and CRANN/AMBER

Trinity Translational Medicine Institute (TTMI)

Trinity College Dublin

James’s street, Dublin 8, Ireland

Tel. +353 1 896 3259

Email: prinamea@tcd.ie

**Ag+ collection and quantification**

For the quantification of the Ag^+^ percentage for each preparation, a slight modification of the standard HF5 setup was designed, to improve the efficacy of ion collection. Therefore, a T-valve was added in order to deviate the focus flow and reduce dead volumes during the focus-injection step devised (Method in Table 1). The applicability of the method was demonstrated in previous works [30] and confirmed through injection, collection and quantification of known amounts of AgNO_3_. The procedure is schematised in Figure S1.


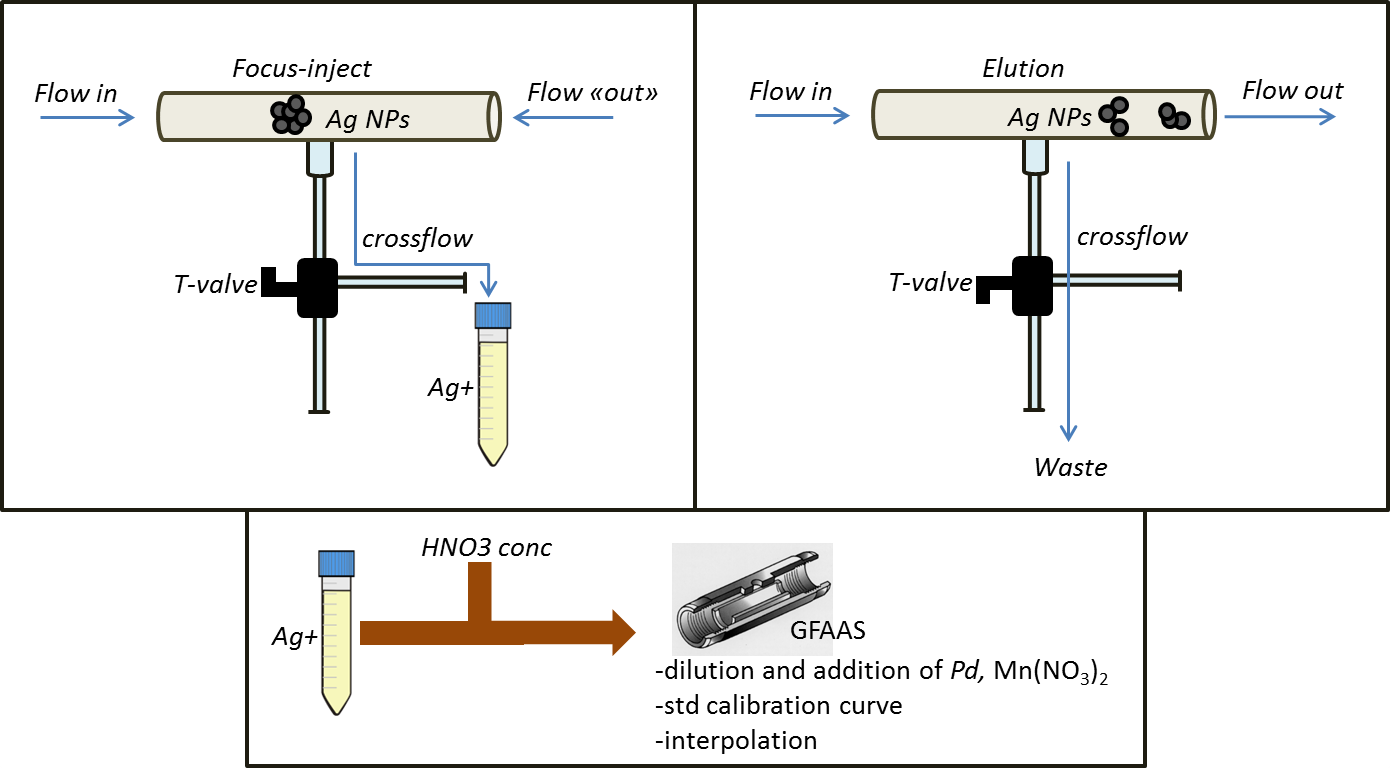


**Figure S1.** Schematic procedure of ionic Silver fraction collection and quantification

**HF5 separation and characterisation**

The LS @90° recorded on line during the separation is available in Figure S2, together with the R_g_ distribution calculated by MALS for each peak.

**
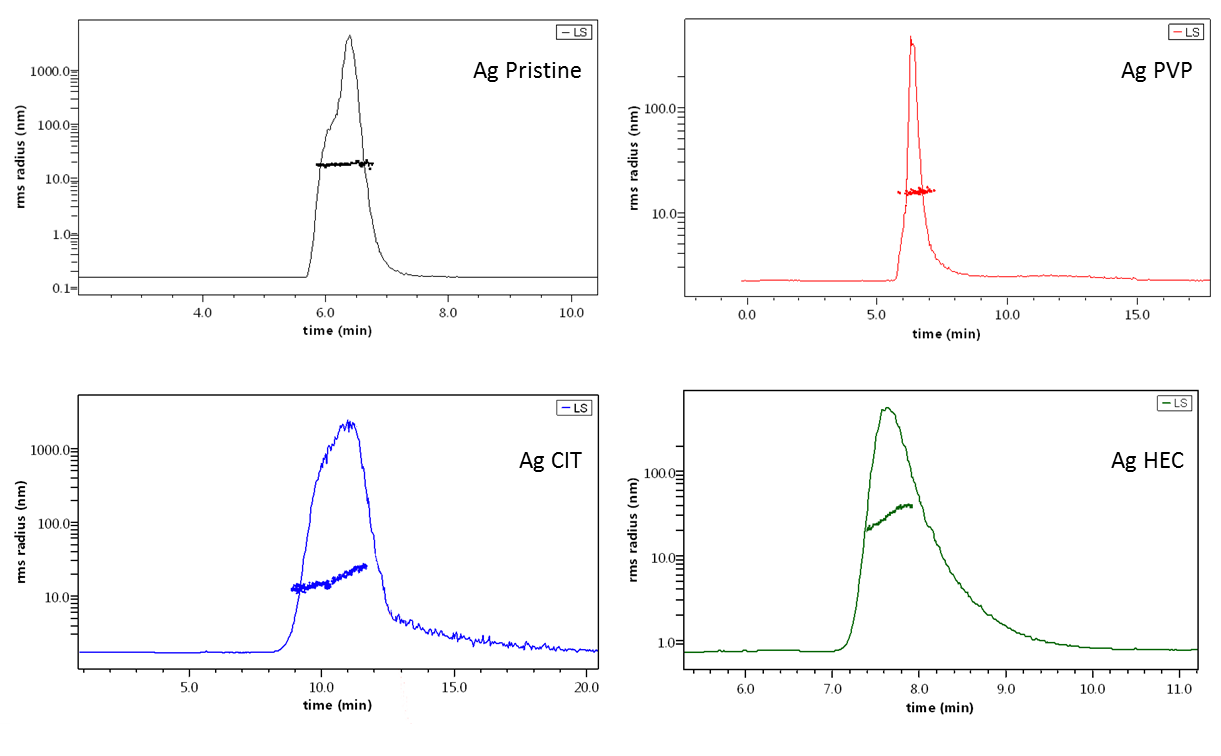
**

**Figure S2.** HF5-MALS signals and R_g_ distribution over time for the four preparation of Ag NPs: Ag Pristine (black), Ag PVP (red), Ag CIT (blue) and Ag HEC (green).

**Cytokine secretion.**

After 24 h of incubation with Ag NPs, the presence of Tumour Necrosis Factor-alpha (TNF-α), Interleukin-6 (IL-6), Interleukin-8 (IL-8) and IL-1β in the culture media of the A431 and HaCAT cells was determined with ELISA Biolegend standard sets (Biolegend, Inc., San Diego, USA). 100 µl of medium were transferred into 96-well plates, previously functionalized overnight with anti-cytokine antibodies, and incubated for 2 hours at room temperature (RT). Then 100 µl of diluted Detection Antibody were added in each well and, after 1 hour of incubation at RT, 100 µl of diluted Avidin-HRP were added. After 30 min, samples were incubated with 100 µl of TMB Substrate Solution, contained in the kit; after 30 min, reaction was stopped and absorbance was immediately read at 450 nm with a plate reader. Standards were performed in the assay diluent from a solution of 50 ng/ml of the recombinant cytokine, as for manufacturer’s protocol.

The results are shown in Figure S3.

For A431 cells, a significant secretion of TNF-α, IL-6 was registered only after the treatment of cells with Pristine and Ag PVP (Figure S3 A-C-E). In HaCaT cells, we detected a statistically significant increase of medium IL-6after exposure to Pristine, Ag PVP and Ag CIT. Ag PVP and Ag CIT induce an increase of IL-8 secretion at higher doses used, whereas TNF-α increase was registered only after exposure to Ag CIT. On the contrary, Ag HEC did not induce any increase of the three cytokines in the culture medium (Figure S3 B-D-F). However, for all the four preparations of Ag NPs, and for both cell lines, the production of IL-1β in cell culture medium was not significantly induced compared to the control cells (Figure S3 G-H).


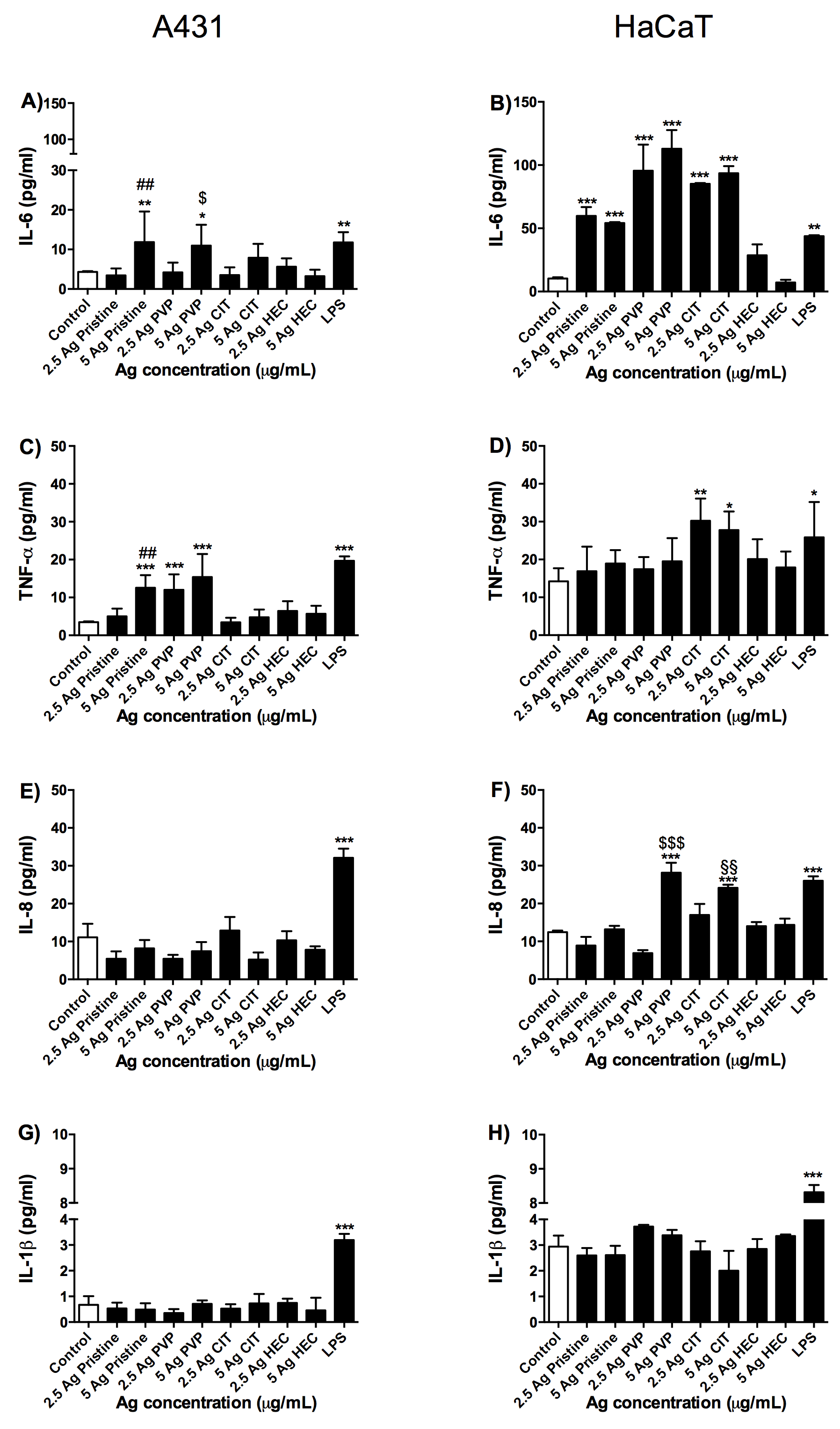


**Figure S3.**  Recovery A431 and HaCaT cells after exposure to Ag NPs. Cells, grown for 24 hours in complete growth medium, were treated with different concentrations of Ag NPs or with ethanol (80%), used as positive control. After 24h of exposure cell medium was replaced with full growth medium and cells were cultured for 6 additional days. On the seventh day viability was assessed using calcein assay. (A), (C), (E) and (G): A431; (B), (D), (F) and (H): HaCaT. Data are means of 3 independent determinations ± SD. * p<0.05, **p<0.01 and *** p<0.001 vs. untreated, control cells. Ag Pristine (A-B), Ag PVP (C-D), Ag CIT (E-F) and Ag HEC (G-H).
